# Artificial Intelligence in the Management of Breast Cancer: A Comprehensive Review

**DOI:** 10.7759/cureus.106764

**Published:** 2026-04-10

**Authors:** Soufia El Ouardani, Hind Chibani, Farah El Ouardani, Sami Aziz Brahmi, Said Afqir

**Affiliations:** 1 Medical Oncology, Mohammed VI University Hospital, Oujda, MAR; 2 Medical Oncology, Faculty of Medicine and Pharmacy of Oujda, Mohamed First University, Oujda, MAR; 3 Biology, Multidisciplinary Faculty of Nador, Mohamed First University, Nador, MAR

**Keywords:** artificial intelligence, breast cancer, convolutional neural networks (cnn), deep-learning, machine leaning, robotic surgery

## Abstract

Artificial intelligence (AI) has rapidly emerged as a transformative tool in modern medicine, particularly in oncology, where it has shown significant potential in improving diagnostic accuracy and early detection of cancer. Breast cancer remains one of the most prevalent malignancies worldwide, and early diagnosis is crucial for reducing mortality.

This review provides a comprehensive overview of the current applications of AI in cancer diagnosis, with a specific focus on breast cancer. A narrative review of recent literature was conducted using major databases such as PubMed and Scopus, including studies on machine learning and deep learning techniques applied to imaging, histopathology, and clinical decision-making. AI-based models, particularly convolutional neural networks, have demonstrated high accuracy in analyzing mammographic images and detecting early-stage breast cancer, with some studies showing performance comparable to, or exceeding, that of experienced radiologists. Furthermore, AI has contributed to improved lesion classification, reduced false-positive rates, and enhanced diagnostic efficiency. In histopathology, AI systems have also shown strong capabilities in tumor detection and grading. Despite these promising advancements, several challenges remain, including data bias, lack of standardization, ethical concerns, and limited integration into clinical practice. Overall, AI represents a promising approach for improving breast cancer diagnosis, although further large-scale validation and clinical implementation are needed.

## Introduction and background

Breast cancer continues to represent a major global health burden, requiring complex and multidisciplinary management strategies that span early detection, accurate diagnosis, treatment planning, and long-term follow-up [1]. In this context, artificial intelligence (AI) has emerged as a transformative tool with the potential to enhance clinical decision-making and optimize patient outcomes across the entire care pathway [2]. Within breast cancer management, AI is not limited to improving technical performance but increasingly supports integrated and data-driven clinical workflows [3]. In screening programs, AI-assisted mammography has demonstrated the ability to improve detection rates while reducing inter-observer variability among radiologists. By rapidly analyzing high-dimensional imaging data, AI systems contribute to earlier identification of suspicious lesions, which is critical for improving prognosis [4,5].

At the diagnostic level, AI facilitates more precise characterization of breast lesions by extracting quantitative imaging features that may not be perceptible to the human eye. These approaches, often referred to as radiomics, enhance the differentiation between benign and malignant findings and support risk stratification [6]. In parallel, AI applications in digital pathology enable automated analysis of histological samples, improving tumor classification, grading, and biomarker assessment, which are essential for guiding therapeutic decisions. AI also plays a pivotal role in treatment planning and personalization [7]. By integrating clinical, imaging, and molecular data, machine learning models can predict disease progression, treatment response, and patient outcomes [8]. This supports clinicians in selecting optimal therapeutic strategies, including surgery, chemotherapy, radiotherapy, and targeted therapies [8]. Furthermore, AI-based tools are increasingly used to optimize radiotherapy planning and to assist in surgical decision-making, contributing to more precise and individualized interventions [9]. Beyond initial treatment, AI contributes to longitudinal patient management through outcome prediction and monitoring. Predictive analytics can identify patients at higher risk of recurrence and support tailored follow-up strategies. Additionally, AI-driven systems may improve healthcare efficiency by streamlining workflows and reducing diagnostic and therapeutic delays [3]. Despite these advances, the integration of AI into routine clinical practice remains challenging due to issues related to data heterogeneity, model interpretability, and regulatory and ethical considerations. Nonetheless, its expanding role highlights a shift toward more precise, personalized, and data-driven breast cancer management [2].

This review aims to examine the current role of AI in breast cancer management, focusing on its contributions from detection to treatment and follow-up while addressing key challenges and future directions.

## Review

Methods

This comprehensive review aimed to synthesize the current evidence regarding the role of AI in breast cancer management. A systematic literature search was conducted using major electronic databases, including PubMed, Scopus, and Google Scholar, to identify relevant studies. The search strategy combined controlled vocabulary terms and free-text keywords, such as “artificial intelligence,” “machine learning,” “deep learning,” “breast cancer,” “screening,” “diagnosis,” “prognosis,” “treatment,” and “follow-up,” using Boolean operators to refine the results.

Articles published in English between 2020 and 2025 were considered in order to capture the most recent technological advances and clinical applications of AI. Eligible studies included original research articles, systematic reviews, and meta-analyses that specifically investigated the use of AI-based methods in any aspect of breast cancer care. Studies were included if they evaluated the performance, clinical utility, or implementation of AI tools in areas such as imaging, risk prediction, therapeutic decision-making, or patient monitoring. In contrast, studies were excluded if they did not involve AI methodologies, were not focused on breast cancer, or consisted solely of expert opinions without supporting data.

Following the initial screening of titles and abstracts, full-text articles were assessed for eligibility based on predefined inclusion and exclusion criteria. Relevant data were extracted from the selected studies and synthesized according to the main areas of application of AI in breast cancer management, including screening, diagnosis, prognosis, treatment decision-making, and follow-up. Particular attention was also given to study design, type of AI model, and reported performance outcomes.

Discussion

Emergence of AI in Breast Cancer Care

AI has progressively transformed the field of oncology by enabling the analysis of complex and high-dimensional medical data. Its initial applications in cancer research emerged with the development of machine learning algorithms designed to improve pattern recognition in imaging and clinical datasets [2]. Over time, advances in computational power and the advent of deep learning have expanded the role of AI from experimental settings to practical clinical applications [3]. In breast cancer, one of the most extensively studied malignancies, AI was first introduced primarily in imaging, particularly in computer-aided detection (CAD) systems for mammography [3,6]. These early systems aimed to assist radiologists in identifying suspicious lesions, laying the foundation for more sophisticated, AI-driven approaches that now span the entire continuum of care [10].

AI in Breast Cancer Screening and Imaging

In the context of breast cancer screening, AI has demonstrated a significant impact on improving early detection. Mammography remains the cornerstone of screening programs, but its sensitivity can be limited, particularly in women with dense breast tissue [4]. Several studies have demonstrated the effectiveness of convolutional neural networks (CNNs) in mammography for automated lesion detection and classification. For instance, Wang highlighted the ability of deep learning models to analyze mammographic images by learning hierarchical features, enabling accurate detection of microcalcifications and masses while reducing observer variability [10]. Similarly, Jiménez-Gaona et al. provided a critical review of CAD systems based on deep learning, emphasizing their improved sensitivity and specificity compared to traditional CAD approaches [11].

More recent studies have focused on enhancing model performance through advanced architectures and large-scale datasets. Carriero et al. described state-of-the-art deep learning models, including CNNs and hybrid architectures, capable of performing multiple tasks, such as detection, segmentation, and classification, thereby supporting comprehensive diagnostic workflows [4]. In addition, multimodal AI approaches have emerged as a promising direction. A density-informed framework integrates imaging data with breast density information to improve detection accuracy across different breast tissue types, addressing a major limitation of conventional mammography [12]. Similarly, the Deep-LIBRA model, developed by Haji Maghsoudi et al., enables automated and robust quantification of breast density, which is a critical factor in both cancer detection and risk stratification [13].

Beyond detection, radiomics-based AI approaches have further expanded the role of imaging in oncology. Papadimitroulas et al. highlighted how radiomics, combined with machine learning, can extract high-dimensional quantitative features from medical images, providing valuable insights for tumor characterization and supporting personalized decision-making [2]. In a broader perspective, Ali et al. reviewed various AI techniques applied to breast cancer imaging, confirming their ability to enhance diagnostic accuracy, reduce false positives, and improve workflow efficiency [5].

At the diagnostic stage, AI contributes to a more precise and standardized evaluation of breast lesions. In radiology, AI-driven tools analyze imaging features quantitatively through radiomics, extracting data related to shape, texture, and intensity that are not readily visible to the human eye [2]. These features can be used to differentiate benign from malignant lesions and to predict tumor aggressiveness. AI also improves consistency in image interpretation, reducing inter-observer variability among radiologists [14]. In breast ultrasound, deep learning models assist in automated lesion segmentation, classification, and characterization. They provide measurements of size, shape, and vascularity that help radiologists assess malignancy risk, monitor treatment response, and predict lymph node metastasis. This, in turn, enhances preoperative assessment and clinical decision-making [15,16]. Similarly, in breast MRI, AI algorithms can analyze multiparametric sequences to evaluate lesion morphology, enhancement kinetics, and tumor extent, supporting accurate staging and surgical planning [17]. Additionally, AI integration into diagnostic workflows allows for faster image processing and decision support, facilitating timely clinical interventions.

At the metastatic evaluation stage, AI has demonstrated a significant role in enhancing the detection and characterization of metastatic lesions in breast cancer. On computed tomography (CT) imaging, deep learning algorithms can automatically identify nodules in the lungs, lymph node enlargements, and hepatic lesions, improving sensitivity and specificity, particularly for small or subtle metastases that may be overlooked by human observers [18,19]. These models can also segment lesions and quantify their size and volume, providing objective metrics for disease monitoring and treatment response assessment. In positron emission tomography (PET)/CT imaging, AI-assisted tools enable automated analysis of metabolic activity, including standard uptake values (SUV), allowing precise identification of hypermetabolic areas suggestive of metastatic involvement in bones, lymph nodes, and visceral organs [20,21]. Overall, AI integration in metastatic imaging facilitates earlier detection, standardized reporting, and more accurate monitoring of disease progression, ultimately contributing to personalized management strategies in breast cancer care.

AI in Breast Biopsy and Histopathology

Biopsy procedures, although already highly standardized, can also benefit from AI integration. AI-guided imaging techniques can improve the precision of needle placement by accurately localizing suspicious lesions, particularly in challenging cases [22]. Additionally, AI can assist in selecting the most representative area of the tumor for sampling, thereby reducing sampling errors and improving diagnostic yield. These advancements contribute to a more reliable and efficient diagnostic process, which is essential for initiating appropriate treatment [23].

At the diagnostic histopathology stage, AI has increasingly supported pathologists in the evaluation of breast cancer tissue, enabling high-throughput, quantitative, and reproducible analyses. CNNs and other deep learning architectures, applied to whole-slide images, allow automated detection and segmentation of tumor regions, identification of mitotic figures, and analysis of cellular morphology and tissue architecture that are often challenging to assess manually [24,25]. These models can differentiate tumor subtypes, detect subtle morphological patterns associated with aggressive phenotypes, and highlight regions of interest for further review, thereby reducing inter-observer variability and standardizing interpretations across institutions [8].

Beyond structural analysis, AI-based systems also integrate machine learning algorithms, such as random forests and support vector machines (SVMs), to perform quantitative immunohistochemical (IHC) analysis [26]. These systems measure staining intensity, percentage of positive cells, and spatial heterogeneity for markers including estrogen receptor (ER), progesterone receptor (PR), human epidermal growth factor receptor 2 (HER2), Ki-67 proliferation index, and programmed death-ligand 1 (PD-L1), providing objective data crucial for accurate tumor subtyping, prognostic assessment, and therapeutic decision-making [7]. Some AI frameworks combine CNN-based feature extraction with attention mechanisms to focus on diagnostically relevant regions, improving accuracy in heterogeneous tissue samples and identifying high-risk cellular patterns that may predict tumor aggressiveness or likelihood of recurrence [24,26]. Furthermore, AI can assist in integrating histopathological features with clinical, genomic, and imaging data, creating multi-modal predictive models for tumor grading, risk stratification, and therapy response prediction [27].

**Figure 1:**
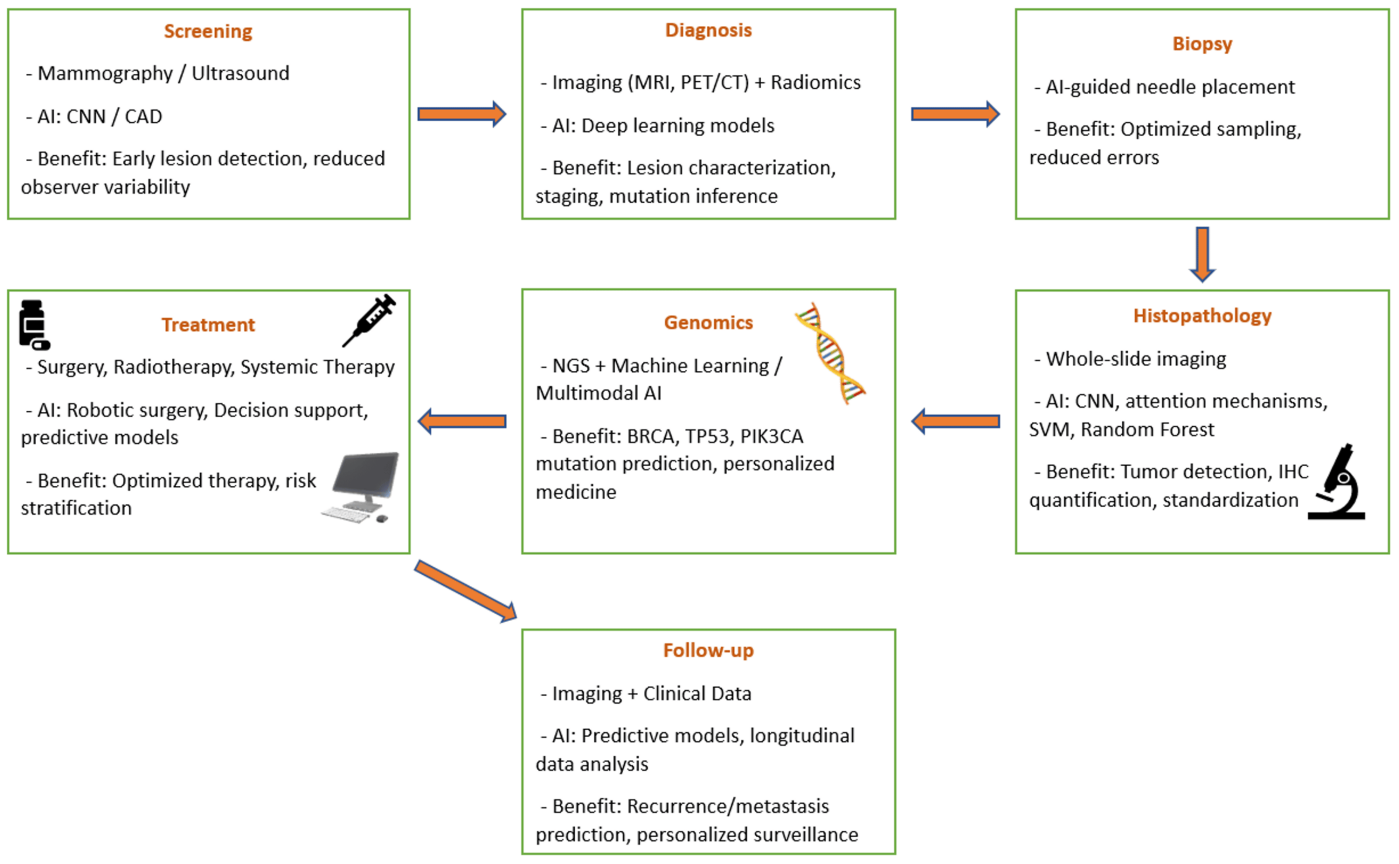
Overview of AI applications across the breast cancer care continuum, from screening and diagnosis to treatment planning and follow-up care. This image is an original, author-created schematic, created using Microsoft PowerPoint 2019 (Microsoft® Corp., Redmond, WA, USA) and was not generated using AI. CNN: convolutional neural network; CAD: computer-aided detection; AI: artificial intelligence; MRI: magnetic resonance imaging; PET/CT: positron emission tomography/computed tomography; SVM: support vector machine; IHC: immunohistochemical

In addition, hybrid models, combining CNNs with attention mechanisms or graph-based neural networks, can integrate histopathological features with clinical and molecular data to predict tumor grade, aggressiveness, and potential response to therapy, thereby enabling a more comprehensive and personalized assessment [28]. Deep learning approaches can also identify morphological patterns associated with specific genomic alterations, including BRCA mutations, through the analysis of histopathological images [29]. Furthermore, radiomics-based methods extract quantitative imaging features from mammography or magnetic resonance imaging to infer underlying molecular characteristics [30]. In parallel, AI algorithms applied to next-generation sequencing data allow accurate detection and classification of genomic alterations, such as TP53 and PIK3CA mutations [29], as illustrated in Figure 1.

AI in Breast Cancer Treatment and Prognosis

In terms of treatment planning, AI plays a central role in advancing personalized medicine in breast cancer care. Machine learning models can integrate diverse datasets, including clinical information, imaging findings, genomic profiles, and histopathological data, to predict treatment response and patient outcomes [8,27]. This enables clinicians to tailor therapeutic strategies to individual patients. In surgical management, AI supports decision-making between breast-conserving surgery and mastectomy, assists in preoperative planning, and is increasingly integrated into robotic surgery systems to enhance precision and optimize resection of both primary tumors and selected metastatic lesions [31,32]. Although AI in breast surgery remains largely exploratory during the intraoperative phase and is currently limited to specific tasks, such as instrument tracking, rapid technological advances highlight its strong future potential. In the postoperative phase, AI is more established, improving remote monitoring, early detection of complications, personalized pain management, as well as aesthetic and psychological assessment [33].

In chemotherapy, machine learning models predict treatment response and toxicity profiles based on clinical and molecular data, allowing more personalized regimen selection [34,35]. AI models have been applied to diverse data sources, including clinical records, histopathology images, and molecular profiles, to improve outcome prediction, especially for challenging subtypes such as triple-negative breast cancer (TNBC) and HER2-positive disease. These approaches have demonstrated strong performance in predicting response to neoadjuvant chemotherapy, recurrence risk, and survival outcomes, often outperforming traditional methods. Additionally, AI-driven analyses support the exploration of novel therapies, such as immunotherapy, and enable more precise, individualized treatment strategies [36].

In radiotherapy, AI assists radiation oncologists in tumor segmentation, automated contouring, dose optimization, and minimizing exposure to surrounding healthy tissues, thereby improving treatment accuracy and safety [37]. In hormonal and targeted therapies, AI-driven models analyze receptor status and genomic alterations to guide the selection of endocrine treatments and targeted agents, ensuring a more individualized therapeutic approach [38,39].

Beyond initial treatment, AI contributes significantly to follow-up and prognosis assessment. Predictive models can identify patients at higher risk of recurrence or metastasis, allowing for more personalized surveillance strategies [8]. AI-driven systems can also monitor treatment response through longitudinal analysis of imaging and clinical data, enabling early detection of disease progression or relapse [35]. This dynamic approach to patient management enhances long-term outcomes and supports proactive clinical decision-making (Figure 1).

Challenges and Future Perspectives of AI in Breast Cancer Management

Despite its significant potential, the integration of AI into breast cancer management remains constrained by several challenges, including data heterogeneity, lack of standardization, and the limited interpretability of many models, which may reduce clinical trust. Ethical and regulatory concerns, particularly regarding data privacy and algorithmic bias, further complicate its implementation. Although AI systems often demonstrate strong performance in controlled environments, their generalizability and real-world applicability remain uncertain [11,40]. Emerging approaches, such as standardized multimodal datasets, explainable AI, and privacy-preserving techniques, including federated learning, offer promising solutions to these limitations. Ultimately, AI cannot replace clinical expertise but should be integrated into multidisciplinary workflows, with successful adoption relying on robust validation, improved interoperability, and close collaboration between clinicians, data scientists, and policymakers [33].

## Conclusions

In summary, AI is increasingly embedded in all phases of breast cancer management, from screening and diagnosis to treatment and follow-up. Its ability to process large volumes of complex data and generate clinically relevant insights positions it as a key driver of precision medicine. While challenges remain, ongoing advancements and validation studies are likely to further consolidate the role of AI as an indispensable tool in modern breast-cancer care.
